# Tick-borne zoonoses in the Order Rickettsiales and Legionellales in Iran: A systematic review

**DOI:** 10.1371/journal.pntd.0006722

**Published:** 2018-09-11

**Authors:** Faham Khamesipour, Gabriel O. Dida, Douglas N. Anyona, S. Mostafa Razavi, Ehsan Rakhshandehroo

**Affiliations:** 1 Cellular and Molecular Research Centre, Sabzevar University of Medical Sciences, Sabzevar, Iran; 2 Department of Pathobiology, School of Veterinary Medicine, Shiraz University, Shiraz, Iran; 3 School of Public Health and Community Development, Maseno University, Maseno, Kenya; 4 Department of Community and Public Health, Technical University of Kenya, Nairobi, Kenya; 5 School of Environment and Earth Sciences, Maseno University, Maseno, Kenya; Baylor College of Medicine, UNITED STATES

## Abstract

**Background:**

Tick-borne zoonoses in the Order Rickettsiales and Legionellales cause infections that often manifest as undifferentiated fevers that are not easy to distinguish from other causes of acute febrile illnesses clinically. This is partly attributed to difficulty in laboratory confirmation since convalescent sera, specific diagnostic reagents, and the required expertise may not be readily available. As a result, a number of tick-borne zoonoses are underappreciated resulting in unnecessary morbidity, mortality and huge economic loses. In Iran, a significant proportion of human infectious diseases are tick-borne, with anecdotal evidence suggesting that tick-borne zoonoses are widespread but underreported in the country. Epidemiological review is therefore necessary to aid in the effective control and prevention of tick-borne zonooses in Iran. The aim of this review is to provide an in-depth and comprehensive overview of anaplasmosis, ehrlichiosis, spotted fever group rickettsioses and coxiellosis in Iran.

**Methods:**

Using the Preferred Reporting Items for Systematic Reviews and Meta-Analyses (PRISMA) guidelines, all relevant publications on tick-borne zoonoses in the Order Rickettsiales and Legionellales in Iran were searched using a number of search terms. The search was confined to authentic resources from repositories of popular data bases among them PubMed, Web of Science, Google Scholar, Science Direct, SpringerLink and SCOPUS. The search items included peer reviewed journals, books and book chapters published between 1996 and 2017.

**Results:**

A total of 1 205 scientific publications and reports were sourced, of which 63 met the search criteria and were reviewed. Of the 63 articles reviewed, 36 (57.1%) reported on coxiellosis, 15 (23.8%) on anaplasmosis, 11 (17.5%) on ehrlichiosis and 1(1.6%) on spotted fever group rickettsiae in a large scale study involving four countries, among them Iran. The existence of tick-borne pathogens in the Order Rickettsiales and Legionellales was confirmed by molecular, serological and microscopic techniques conducted on samples obtained from sheep, cattle, goats, camels, poultry, animal products (milk and eggs), dogs, ticks and even human subjects in different parts of the country; pointing to a countrywide distribution.

**Discussion:**

Based on the review, coxiellosis, anaplasmosis, ehrlichiosis, and SFG rickettsiae can be categorized as emerging tick-borne zoonotic diseases in Iran given the presence of their causiative agents (*C*. *burnetii*, *A*. *phagocytophilum*, *A*. *marginale*, *A*. *bovis*, *A*. *ovis*, *A*. *central*, *E*. *canis*, *E*. *ewingii*, *E*. *chaffeensis* and *R*. *conorii*) collectively reported in a variety of domestic animals, animal products, arthropods and human beings drawn from 22 provinces in Iran.

**Conclusion:**

Given the asymptomatic nature of some of these zoonoses, there is a high likelihood of silent transmission to humans in many parts of the country, which should be considered a public health concern. Presently, information on the transmission intensity of tick-borne zoonoses caused by pathogens in the Order Rickettsiales and Legionellales to humans and its public health impact in Iran is scanty.

## Author summary

Tick-borne zoonoses caused by pathogens in the Order Rickettsiales and Legionellales are significant causes of morbidity and mortality in many parts of the world. In recent times, incidences of zoonotic diseases have increased due to the changing climate, cross-border movement of tick-infested animals as well as advancement in molecular techniques that have aided their rapid identification. Tick-borne zoonoses infect and multiply in most organs of ticks, particularly the salivary glands, thus enabling pathogen transmission to a variety of hosts among them mammals, birds, reptiles and amphibians mostly through the bite of an infected tick. These tick-borne zoonoses are therefore geographically localized and occur mostly in foci with optimal conditions for the vectors thus posing a public health concern. Like in many regions across the world, tick-borne zoonoses; caused by pathogens in the Order Rickettsiales and Legionellales, have not received the level of public attention that has been paid to other maladies in Iran. As a result, their presence and actual magnitude is still under-reported in the country; necessitating this systematic review. Sixty three publications were reviewed after the initial evaluation of 1,205 articles. Most of the 63 publications reported on coxiellosis in Iran followed by anaplasmosis, ehrlichiosis and SFG rickettsiae in that order. Pathogens causing these diseases and their antibodies were collectively detected in domestic animals (sheep, cattle, goats, camels and dogs), animal products (milk and eggs), arthropods (ticks) and human beings in 22 of the 31 provinces in Iran. People in close contact with domestic animals and animal products like farmers, veterinarians, slaughterhouse workers, laboratory personnel and health care workers are at higher risk of infection. Findings emanating from this review suggest that tickborne zoonoses caused by pathogens in the Order Rickettsiales and Legionellales could be prevalent in parts of Iran though the studies are insufficient to give the general epidemiology of the zoonoses in Iran. Besides, the existing studies only covered a limited number of organisms at small spatial scales thus excluding a large portion of the country and a whole range of other potential reservoirs such as horses, pigs, cats, donkeys and wildlife thus creating gaps in knowledge that need to be filled.

## Introduction

Tickborne zoonoses caused by pathogens in the Order Rickettsiales and Legionellales are significant sources of morbidity and mortality in many countries and are increasingly being recognized across the world [1]. Incidence of tick-borne zoonoses has been on the increase in recent times and this could be attributed to the changing climate and cross border movement of tick-infested animals. Besides, development of new techniques and advancement of molecular techniques has facilitated accurate identification and assignment of correct phylogenetic positions to many pathogens that cause tick-borne zoonoses [1]. Over the last decade, the taxonomy of Order Rickettsiales has undergone extensive reorganization [1,2]. *Coxiella burnetii*, the agent of Q fever; previously designated as a *Rickettsia*, has now been re-classified as a Gamma-proteobacteria in the Order Legionellales, leaving the Order Rickettsiales with two families: *Anaplasmataceae* and *Rickettsiaceae* [1]. The Order *Rickettsiales* now covers seven main genera, namely: *Anaplasma*, *Ehrlichia*, *Midichloria*, *Neorickettsia*, *Orientia*, *Rickettsia* and *Wolbachia*, while Order Legionellales comprises of two families: Legionellaceae that is made up of *Legionella*, *Fluoribacter* and *Sarcobium* and Coxiellaceae that is made up of *Coxiella*, *Aquicella* and *Rickettsiella* species [1]. Some of these genera include notable pathogens like *Coxiella burnetii*, *Anaplasma phagocytophilum*, *A*. *maginale*, *Ehrlichia chaffeensis*, *E*. *ewingii* and *E*. *canis* among others [3]. Apparently, classification of the different Orders of tick-borne zoonoses continues to be modified as new data becomes available [1].

A number of tick-borne zoonoses caused by pathogens in the Order Rickettsiales and Legionellales have been reported worldwide with their ecology being influenced by environmental factors and availability of specific vectors that determine their establishment and epidemiology. By the 20^th^ century for instance, only three rickettsioses were recognized in America and these included: Rocky Mountain Spotted Fever (RMSF), epidemic typhus and endemic typhus. However, since 2000, more than 10 different rickettsial species previously unknown have been described in arthropods and in clinical cases [4]. Studies also show that the period between 1984 and 2005 witnessed the identification of 11 additional rickettsial species or sub-species as emerging agents of tick-borne rickettsiales across the world [5].

Overwhelming evidence shows that tick borne pathogens in the Order Rickettsiales and Legionellales are prevalent in the Middle East region including Iran. For instance, records of cases reported to the African Union–Inter African Bureau for Animal Resources (AU-IBAR) in 2011, showed that anaplasmosis was prevalent in a number of countries in the Middle East including Iran, Iraq, UAE, Egypt, Qatar, Cyprus, Israel and Jordan [6, 7, 8, 9, 10]. In addition, a number of *Ehrlichia* spp., *Anaplasma* spp. and SFG rickettsial pathogens as well as *Coxiella burnetii* pathogens have been detected among different organism in a number of countries within the Middle East. Besides, widespread distribution of Ixodid ticks points to the existence of many natural foci in the region as well as in Iran [11]. Epidemiology of the specific diseases is the Middle East and Iran is highlighted in the respective sub-sections below.

### Coxiellosis

*Coxiella burnetii* is a zoonotic and strictly intracellular Gram-negative bacterium that belongs to the Gammaproteobacteria, and is the agent of Q fever [12]. The main reservoirs of *C*. *burnetii* are cattle, sheep, and goats. However, in recent years, an increasing number of animals have been reported to shed the bacterium, including domestic mammals, reptiles, marine mammals, ticks, and birds [13]. While birth products contain the highest concentration of the bacteria, *C*. *burnetii* is also found in urine, feces and milk of infected animals [14]. The feces of ticks infected with *C*. *burnetii* have particularly high concentrations of viable organisms capable of persisting for relatively longer periods in the environment, and as such, ticks play a crucial role in the circulation of the pathogen [15].

Transmission to human most frequently occurs through inhalation of aerosolized bacteria that are spread in the environment by infected animals after delivery or abortion. Such windborne outbreaks can affect dozens to hundreds of people who may not have direct exposure to the infected animals. Varying incidence levels of Q fever have been reported globally since the disease was first discovered in 1937 in Australia [16]. In the United States, Q fever became a reportable disease in 1999 after which an increase of 250% was reported in the number of human cases between 2000 and 2004. This was attributed to better recognition of cases [17]. According to CDC [13] twenty seven EU/EEA countries provided information on Q fever in 2014. A total of 822 cases were reported to The European Surveillance System (TESSy), 782 of which were confirmed (95.1%). Most of the cases were reported in Germany (262, 90.1% of which were confirmed) and France (209, all confirmed). Between 2007 and 2010, more than 4,000 human cases (with 14 deaths) were reported in Netherlands in the largest Q fever epidemic ever to be reported in that country. Kaplan and Bertagna [18] reported initial cases of Q fever in nine African countries in 1955. Seroprevalence in humans ranged between 1 and 32% in Chad, Egypt, Coˆte d’Ivoire, Burkina Faso and Dar es Salaam Tanzania [19].

Coxiellosis has been recognized as being endemic in a number of countries in the Middle East region. In Cyprus, coxiellosis was recognized since 1951 [18, 20, 21, 22, 23], while in Israel, the first proven case of Q fever (75 cases in Haifa area) was reported in 1949 [24]. Subsequently, Q fever outbreaks have been reported in Iraq, Israel, Turkey, Saudi Arabia and Egypt with prevalence rates ranging between 0% and 90% among different organisms in different countries [25]. In Iran; also within the Middle East, the first clinical case of acute coxiellosis was reported in 1952, with many more cases being reported between 1970 and 1976 in different countries in the Middle East region [26]. After the 70s, coxiellosis remained neglected in Iran until 2007 when *C*. *burnetii* antibodies were reported among febrile patients in Kerman province, southeastern Iran [27]. Since then, many studies have been conducted among different organisms including human subjects and shown that the disease is endemic in Iran [28]. Nevertheless, the information available is scattered, disjointed and largely insufficient to give the overall prevalence of Q fever in Iran, necessitating this systematic review.

### Anaplasmosis

*Anaplasma* species of the family Anaplasmataceae, Order Rickettsiales are tick-borne pathogens that can cause a number of diseases in animals and humans. Six species; *Anaplasma ovis*, *A*. *marginale*, *A*. *centrale*, *A*. *platys*, *A*. *bovis* and *A*. *phagocytophilum* are well recognized [29, 30]. These species are obligate intracellular bacteria that parasitize erythrocytes and monocytes of higher vertebrates; mostly ruminants and are particularly important among livestock breeders. *Anaplasma platys* is mainly pathogenic to canines [31], while *A*. *ovis* mainly affect goats, cattle and sheep. *Anaplasma marginale*, *A*. *bovis*, and *A*. *centrale* are well recognized in cattle but also cause sub-clinical or mild conditions in other domestic animals [32]. *Anaplasma phagocytophilum* is the etiologic agent of Human Granulocytic Anaplasmosis (HGA) but also affects a number of animals such as horses, cattle, sheep, goats, dogs and cats. Small rodents also harbor different *Anaplasma* spp. and thus act as potential reservoirs [33].

Anaplasmosis is widely distributed throughout the world including tropical and sub-tropical areas of South, Central and North America, Australia, Asia and Europe [34], with a prevalence ranging between 1–100% [35]. A number of anaplasmosis causing pathogens to have been reported in different countries including: United States [36], Venezuela [37], Cyprus [38], China [39], Spain [40], among many others. In the United States of America, anaplasmosis has been reported in almost every state; a phenomenon attributed to cross border movement of carrier cattle and subsequent mechanical or biological transmission of the pathogens to susceptible livestock [41]. In all Latin America countries and the Caribbean Islands, anaplasmosis is enzootic with the exception of desert areas and certain mountain ranges (Andes) [42]. In Africa, outbreaks of bovine anaplasmosis have been reported in different countries including Kenya, Tanzania, Morocco, Uganda, Ghana among others [43]. In Europe, granulocytic anaplasmosis is the most widespread tick-borne infection in animals [44] and both its geographic distribution and that of its vector (*Ixodes ricinus)* is increasing in latitude and altitude [45, 46].

In the Middle East, *Anaplasma* spp. seems to be prevalent in the region; going by the available studies. Records contained by the AU-IBAR, show that anaplasmosis was reported in a number of countries among them Iraq, UAE, Egypt, Qatar, Cyprus, Israel and Jordan as at 2011 [6, 7, 8, 9]. However, the report also revealed that many other countries from the Middle East region did not have any study reporting on anaplasmosis nor did it have any reported cases of *Anaplasma* spp. Nevertheless, a number of independent studies have reported cases of various *Anaplasma* spp. in different organisms in the region. For instance, in a study described as the first ever among cattle in Turkey, *A*. *phagocytophilum* was reported as the most prevalent (30.8%) followed by *A*. *marginale* (18.8%), *A*. *centrale* (18%), and *A*. *bovis* (1/133, 0.7%) in that order [47]. *A*. *phagocytophilum* has also been detected in goats and sheep in Cyprus, and also in ticks in Israel [48], while *Anaplasma platys* has been reported among dogs in Cyprus and many other countries [49].

Since its’ first description in Palestine in the early 1920s by Gilbert [50], *A*. *ovis* has been reported among different animals in Turkey [51], Cyprus [52], Jordan [53], Iraq [54] and many other countries in the Middle East. Likewise, *A*. *centrale* has also been reported in different countries in the Middle East [55]. In Iran, five species in the genus *Anaplasma* namely: *A*. *ovis*, *A*. *bovis*, *A*. *marginale*, *A*. *centrale* and *A*. *phagocytophilum* have been isolated from cattle, sheep, goats, ticks and human serum samples; though most studies on anaplasmosis are limited to a section (northern part) of the country. From the foregoing, it is evident that *Anaplasma* spp. pathogens are circulating in a number of animal reservoirs in Iran making tickborne zoonoses highly endemic in the country. However, studies on the different pathogenic species are patchily distributed among the countries of the Middle East region, making it difficult to establish the actual prevalence of anaplasmosis in the region.

### Ehrlichiosis

Ehrlichiosis comprises of a group of emerging infectious tick-borne zoonoses that are caused by obligate intracellular Gram-negative bacteria in the genus *Ehrlichia*, family Anaplasmataceae, Order Rickettsiales [56]. Several species of *Ehrlichia* such as *E*. *chaffeensis*, *E*. *muris*, *E*. *ewingii*, *E*. *canis*, *E*. *equi*, *E*. *ruminantium* and *E*. *risticii* are known to infect a number of animals with *E*. *canis* being the main species that infects dogs; producing several clinical symptoms [57]. Apart from dogs, *Ehrlichia* spp. are also known to cause illness not only in human beings but also in other animals like cattle, sheep, goats, horses, dogs, deer, rodents and mice [58, 59].

Human Monocytic Ehrlichiosis (HME) that is caused by *Ehrlichia chaffeensis* was first described in 1986, and after about 2 decades, more than 2,300 cases had been reported to the CDC [60]. This makes it the most prevalent life-threatening tick borne disease in the US where the disease has been reported in the south-central, southeastern and Mid-Atlantic States [60, 61]. Apparently, the occurrence of the disease corresponds to regions where the white-tailed deer *(Odocoileus virginianus)* and lone star ticks (*Amblyomma americanum)* exist. *Ehrlichia chaffeensis* has also been detected in Africa [62].

*Ehrlichia ewingii* that has recently been associated with human infection [63] is prevalent in North America but has also been detected in South America and Africa in recent times. Only a few human cases have been documented, in the USA; mainly in Tennessee, Missouri and Oklahoma. However, *E*. *ewingii* infection in other animals like deer, dogs and ticks has been reported throughout the geographical range of the lone star tick; suggesting that human infection with this pathogen might be more widespread than previously thought [64].

Canine Monocytic Ehrlichiosis (CME) that is caused by *E*. *canis* has a worldwide distribution with varying seroprevalence rates being reported in different countries. CME has been reported in the United States and in many other countries among them Cameroon with a prevalence of 32%, [65], Mexico with 44% [66], Grenada with 24% [67], South Africa with 3%, Zimbabwe with 52% [68] and Brazil with 2–6% [69]. However, no reports show serological presence of CME in Australia.

*Ehrlichia muris* was first detected in 2009 among 3 symptomatic patients in Wisconsin and 1 in Minnessota USA and has also been found in *I*. *persulcatus* complex ticks in Minessota, Wisconsin, Eastern Europe and Japan suggesting that this species may be a potential vector [70, 71]. Equine Granulocytic Ehrlichiosis (EGE) is a disease of horses caused by rickettsial bacteria in the genus *Ehrlichia*. Two Ehrlichia species that are known to infect horses include *E risticii* which infects monocytes and *E equi* that infects granulocytes and is transmitted by the tick *Ixoides pacificus* [72]. Equine granulocytic ehrlichiosis was first reported among horses in northern California by Gribble in 1969 [73]. Since then, the disease has been reported in Minnesota, Wisconsin, Colorado, Illinois, Florida, Washington, Connecticut, New York and New Jersey in the United States, as well as in Germany, Switzerland, Sweden, and Israel [74, 75, 76].

In the Middle East region, outbreaks of canine monocytic ehrlichiosis have been reported in countries such as Saudi Arabia [77], while limited studies on CME have been conducted in other countries like Turkey. A few reports have also documented seropositivity [78], clinical case treatment [79] and molecular prevalence [80] of CME in Turkey, while a CME prevalence of 17–26% was reported in Israel [81]. In addition, cases of Equine Granulocytic Ehrlichiosis (EGE) have also been reported in Israel [74, 75, 76]. Despite being an emerging disease in humans and animals, ehrlichiosis, is not as extensively studied in the Middel East countries as it ought to be [82]. In Iran, *Ehrlichia* species have been detected circulating among dogs and ticks in 12 provinces, most of them located to the northern part of the country. This points to the presence of *Ehrlichia* spp. in the country, thus posing a risk of transmission to humans.

### Spotted Fever Group Rickettsioses

Spotted Fever Group (SFG) Rickettsioses comprises several divergent lineages including: *Rickettsia rickettsii* group, *R*. *japonica*, *R*. *montana*, *R*. *massiliae* group, *R*. *helvetica*, *R*. *felis*, and *R*. *akari* group [2]. SFG rickettsioses occur worldwide and may cause serious diseases in humans. They are transmitted to people by arthropod vectors, such as ticks, fleas, and lice [2]. A number of spotted fever group rickettsial pathogens have been reported across the world. These include: *Rickettsia aeschlimannii*, *R*. *africae*, *R*. *australis*, *R*. *conorii*, *R*. *helvetica*, *R*. *heilongjiangensis*, *R*. *japonica*, *R*. *massiliae*, *R*. *monacensis*, *R*. *raoultii*, *R*. *parkeri*, *R*. *rickettsia*, *R*. *slovaca*, *R*. *sibirica* subsp. *mongolotimonae*, *R*. *honei* and *R*. *marmionii*. Collectively, these rickettsial pathogens were reported in the North, Central and South America, Australia, France, Greece, Spain, Portugal, Switzerland, Argentina, Russia, China, Thailand, Kosovo, Mongolia, India, South Africa, Morocco, Mali, Kenya among other countries [5, 83, 84, 85, 86, 86, 87, 88].

In the Middle East, a number of known species of the genus *Rickettsia* have been documented, though some of the studies are questionable [89]. Al-Deeb et al. [90], reported what they regarded as the first record of a spotted fever group *Rickettsia* spp. in ticks collected from camels in the United Arab Emirates, while *Rickettsia sibrica mongolitimonae* was diagnosed in a male patient in Turkey in 2016 [91]. Mediterranean Spotted Fever (MSF) caused by *Rickettsia conorii* subsp. *israelensi*s and regarded as the primary cause of spotted fever group rickettsiosis in Israel has been detected in ticks collected from roe, deer, addax, red fox and wild boars in the country [92]. In addition, Ereqat et al. [93] detected rickettsial DNA in 148 out of 867 (17%) ticks that were tested in Western Bank, Palestinian Territories, while Chochlakis et al. [94] detected rickettsial DNA in 315 ticks (8%) of the 3,950 ticks screened, in Cyprus. Idris et al. [95] investigated 347 human sera for rickettsial infections in Dhofar, Oman and established that 59% gave positive reactions. The authors concluded that rickettsial infections were common among the rural population of Dhofar in Oman.

Presence of a high variety of ticks which can transmit rickettsiae in several countries including Yemen [96], Saudi Arabia [97] and Oman [98], all within the Middle East region makes it highly likely for the disease to occur in the Middle East region. Despite clear evidence of presence of SFG rickettsial pathogens in the Middle East region, no studies have been conducted and reported on the disease within Iran. It is therefore necessary to initiate studies on SFG Rickettsiae to better understand the epidemiology in Iran.

### Vectors and reservoirs of tickborne rickettsioses and coxiellosis

Ticks are considered second only to mosquitoes as important vectors of human infectious diseases across the world [99]. The obligate hematophagous arthropods parasitize every class of vertebrates in almost every region of the world [15]. For each bacterial disease, one or several tick vectors and one or several reservoirs may exist [100]. Pathogens that transmit tickborne zoonoses rely on a number of ticks in the family Ixodidae (the hard ticks) among them: *Amblyomma*, *Dermacentor*, *Haemaphysalis*, *Hyalomma* and *Rhipicephalus* as vectors and vertebrates such as small mammals, sheep or deer as their reservoir hosts [101].

Only Ixodid ticks are regarded as diseases vectors and pathogens are normally maintained in these ticks by transstadial and transovarial transmission [101]. Tickborne pathogens in the Order Rickettsiales and Legionellales normally infect and multiply in almost all organs of ticks, particularly the salivary glands. This enables ease of transmission of the pathogens from the vectors to the vertebrate hosts through bites of infected ticks during feeding. Transmission can also be through inoculation of infectious fluids or feces from the ectoparasites into the skin. Indirect routes of transmission such as contamination of abraded skin or the eyes following crushing of ticks between the fingers also exist. Infectivity of the reservoir hosts, presence of the tick, infestation rate as well as host density are major variables that determine the epidemiology of tick-transmitted diseases [102].

According to Shemshad et al. [103], a number of Ixodid tick fauna obtained from cattle, sheep, goats, dogs and even shrubbery have been identified in different parts of Iran. The ticks include: *Haemaphysalis concinna*, *H*. *sulcata*, *Hyalomma anatolicum*, *Hy*. *asiaticum*, *Hy*. *detritum*, *Hy*. *dromedarii*, *Hy*. *marginatum*, *Hy*. *schulzei*, *Rhipicephalus bursa*, *R*. *sanguineus*, *Ixodes ricinus* and *Dermacentor* spp. These ticks have the ability to infest a wide variety of hosts including mammals, birds, reptiles and amphibians [104]. Each tick species has preferred environmental conditions and biotopes that determine its geographic range. As such, tick-borne rickettsioses and coxiellosis infections are geographically localized and mainly occur in foci with optimal conditions for the ticks and other animals involved in the circulation of the bacterial pathogens. The presence of a number of Ixodid ticks in Iran therefore implies that there could be many natural foci of tickborne zoonoses in the country from which they may spread to other areas under changing socio-economic and climatic conditions. Despite this fact, tick-borne zoonoses in the Order Rickettsiales and Legionellales have not received the level of public attention that has been paid to other maladies, largely because their presence or the true magnitude of their occurrence is still under reported. Therefore, like in other countries across the world, tick-borne zoonoses are a public health concern in Iran. In recent years, researchers have shown inceased interest on tick-borne zoonotic diseases in Iran. However, the available studies are fragmented and region-specific making it difficult to ascertain the magnitude of these tick-borne diseases in Iran. The aim of this systematic review is to provide an in-depth and comprehensive overview of tick-borne zoonoses in the Order Rickettsiales and Legionellales in Iran.

## Methods

### Focus area of study

The review considered only those publications reporting on tick-borne zoonoses caused by pathogens in the Order Rickettsiales and Legionellales and conducted between 1996 and 2017 in Iran.

### Review design

In this systematic review, the Preferred Reporting Items for Systematic reviews and Meta-Analyses (PRISMA) and guidelines proposed by Arksey and O'Malley [105] were used to select publications and reports on tickborne zoonoses in the Order Rickettsiales and Legionellales in Iran. Using the PRISMA guidelines, published literature on Anaplasmosis, Ehrlichiosis, Spotted Fever Group rickettsiae and Coxiellosis in Iran were systematically searched in PubMed, SpringerLink, SCOPUS, Web of Science, WHOLIS, and U.S Center for Disease Control and Prevention (CDC) databases. To maximize the search and reduce selection bias, the search was restricted to English articles using a number of key terms such as: “Rickettsioses in Iran”; “Rickettsia”; “Tick-borne Rickettsiales in Iran”; “Anaplasmosis”; “Ehrlichiosis”; “vectors of rickettsioses”; “Q fever in Iran”; “Coxiellosis”; “Tickborne Legionellales in Iran” among others. During the initial search, all articles identified from the indexed databases were selected based on their titles and later screened for eligibility based on the content of their abstracts. A full text review of all articles deemed relevant was then conducted.

Data was extracted from the selected publications by filling a table containing six main sections, namely: year of study, disease and study technique used, study region, sample size and organism studied as well as author and year or publication. Another table was used to summarize the data by highlighting the study organism/subject, study area, sample size and author of the article. Disease prevalence was also reported in percentages and their 95% CI provided. The systematic review followed PRISMA guidelines and a PRISMA check list which is provided as a supplementary material (S1 Checklist). The DOI link of the protocol used as a guide in conducting this systematic review can be accessed online through the following link: https://dx.doi.org/10.17504/protocols.io.njbdcin.

### Inclusion and exclusion criteria

Only studies describing findings on anaplasmosis, ehrlichiosis, spotted fever group rickettsioses and coxiellosis infections affecting humans, animals, animal products and arthropods (ticks) in Iran were included in this review. All non-verified sources of information and studies conducted in other parts of the world besides Iran were excluded from this review.

### Risk of bias

This review presents some limitations with regards to missing publications, language bias and publication bias. The combination of terms entered in each individual search aimed at retrieving as many relevant publications as possible but also narrowing down on the amount of results. Hence, it is highly likely that relevant research articles, which did not contain the specified key words in their titles or abstracts, may have been overlooked. In addition, some of the articles retrieved were not written in English and were thus not included in the study, presenting a major bias towards English publications. Furthermore, all the selected publications were obtained through electronic search, thus we acknowledge a bias towards articles published online. Most of the publications included in this review were cross-sectional in design, reporting on anaplasmosis, ehrlichiosis, SFG rickettsioses and coxiellosis infections in a number of organisms at a specific point in time. These types of studies can be subjected to selection and information bias. Another potential source of bias for such studies is the selection of sampled organisms or animals based solely on just their availability (e.g. domestic animals).

## Results

Using the key search terms, a total of 1205 articles among them publications, reports and book chapters were retrieved through internet search using the titles only. Of these, 482 articles were sourced from indexed scientific databases [PubMed, SpringerLink and SCOPUS], while 723 were sourced from generalized searches in Google Scholar, Web of Science, WHOLIS, FAO and CDC databases. The 1205 articles were imported to MS Excel, and 253 articles presenting duplicate titles removed to obtain 952 articles. Further screening was done by title and relevance and a total of 497 articles excluded from the review leaving a sub-set of 455 articles. The 455 articles were further assessed for eligibility by reading through their abstracts. Three hundred and ninty two (392) articles were excluded at this stage following the subject matter suitability leaving 63 eligible articles which underwent full-text assessment; forming the basis for this review (S1 Flow Chart and Table 1). Accession numbers of some of the articles included in this review that were deposited in the gene bank by various authors are also provided as supplementary material (S1 Accession Numbers).

**Table 1:** Summary of reviewed studies on tick-borne zoonoses caused by pathogens in the Order Rickettsiales and legionelalles in Iran (Published between 1996 and 2017).

	Study design / technique used	Year of study	Province	Sample size	Author
**A**	**Coxiellosis**				
1	ELISA test of *Coxiella burnetii* in blood samples	2008	Sistan & Baluchestan	Goats and dairy cattle (n = 169)	[106]
2	ELISA test of *Coxiella burnetii* in blood samples	2009	Kerman	Febrile patients (n = 75)	[107]
3	ELISA test of *Coxiella burnetii* in blood samples	2009	Sistan & Baluchestan	Sheep flocks (n = 85)	[108]
4	ELISA test of *Coxiella burnetii* in milk samples	2010	Kerman	Bulk tank milk samples (n = 44)	[109]
5	ELISA test of *Coxiella burnetii* in blood samples	2010–2011	Mazandaran	Sheep (n = 253)	[110]
6	ELISA test of *Coxiella burnetii* in blood samples	2010–2011	Kerman	Slaughterhouse workers (n = 75)	[111]
7	ELISA test of *Coxiella burnetii* in blood samples	2010–2011	Mazandaran	Sheep (n = 253)	[112]
8	ELISA test of *Coxiella burnetii* in blood samples	2011	Sistan & Baluchestan	Febrile patients (n = 105)	[113]
9	ELISA test of *Coxiella burnetii* in blood samples	2011	Kerman, Homozgan, Sistan & Baluchestan	Goats and sheep (n = 368)	[114]
10	ELISA test of *Coxiella burnetii* in blood samples	2011	Sistan & Baluchestan	Butchers and slaughterhouse workers (n = 190)	[115]
11	ELISA test of *Coxiella burnetii* in blood samples	2011–2012	Ardabil	Sheep (n = 253)	[116]
12	ELISA test of *Coxiella burnetii* in blood samples	2011–2012	Razavi Khorasan, Isfahan, Markazi, Fars	Sheep and goat flocks (n = 180)	[117]
13	ELISA test of *Coxiella burnetii* in blood samples	2011–2012	Kurdistan	Hunters, butchers & health care workers (n = 250)	[118]
14	ELISA test of *Coxiella burnetii* in blood samples	2012	Fars, Isfaham, Markazi & Razavi Khorsan	Sheep and goat flocks (n = 43)	[117]
15	ELISA test of *Coxiella burnetii* in blood samples	2012–2013	North, South and Razavi Khorasan	Camels (n = 167)	[119]
16	ELISA test of *Coxiella burnetii* in blood samples	2013	East Azerbaijan	Febrile patients (n = 116)	[120]
17	ELISA test of *Coxiella burnetii* in blood samples	2014	Hamedan	Sheep (n = 200), goats (n = 50) and cattle (n = 120)	[121]
18	ELISA test of *Coxiella burnetii* in blood samples	2014	Khuzestan and Ardabil	Pregnant women (n = 400)	[122]
19	ELISA test of *Coxiella burnetii* in blood samples	2014–2015	Fars	Asymptomatic companion dogs (n = 181)	[123]
20	ELISA test of *Coxiella burnetii* in blood samples	2014–2015	Mazandaran	Febrile patients (n = 56)	[124]
21	ELISA test of *Coxiella burnetii* in blood samples	NS	Kerman	Veterinary students (n = 121)	[125]
22	ELISA test of *Coxiella burnetii* in blood samples	NS	Khorasan Razavi	Sheep (n = 255) and goats (n = 205)	[126]
23	ELISA test of *Coxiella burnetii* in blood samples	NS	Kerman	Human serum samples (n = 45)	[127]
24	ELISA test of *Coxiella burnetii* in blood samples	NS	Khorasan Razavi	Dairy cattle (n = 246)	[128]
25	ELISA test of *Coxiella burnetii* in blood samples	NS	Tehran	Case report (n = 1) of a 72 year old female	[129]
26	PCR to detect *Coxiella burnetii* in ticks	2009	Kerman	Ticks collected from domestic animals (n = 160)	[130]
27	PCR to detect *Coxiella burnetii*	NS	South Khorsan	Febrile patients (n = 92)	[131]
28	Nested PCR to detect *C*. *burnetii* in eggs	2009–2010	Isfahan, Gilan and Mazandaran	Egg samples from hens, ducks, goose, quails and ostriches (n = 369)	[132]
29	Nested PCR to detect *C*. *burnetii* in in goat milk	2010	Fars, Qom, Kerman, Khuzestan and Yazd	Bulk milk samples (n = 296) from goats	[133]
30	Nested PCR to detect *C*. *burnetii* in dairy bovine milk samples	2011	Qom	Bovine bulk milk samples (n = 100)	[134]
31	Nested PCR to detect *C*. *burnetii* in blood samples	2012–2013	Kerman	Dogs (n = 100)	[135]
32	Nested PCR to detect *C*. *burnetii* in ticks	2014–2015	Sistan & Baluchestan	Ticks (n = 1305)	[136]
33	Nested PCR to detect *C*. *burnetii* in ticks	2014–2015	Sistan & Baluchestan	Ticks (n = 583)	[17]
34	Nested PCR to detect *C*. *burnetii* in ticks	NS	Mazandaran	Hard ticks (n = 2417)	[137]
34	A systematic review (1937 and 2012)	1937–2012	Countrywide	Publications (n = 29)	[3]
35	A systematic review on prevalence of *Coxiella burnetii* (2005–2016)	2005–2016	Countrywide	Publications (n = 28)	[138]
**B**	**Anaplasmosis**				
1	PCR to detect *Anaplasma ovis* and *A*. *marginale* in goats’ blood samples	2008	Golestan and Khorasan Razavi	Goats (n = 170)	[139]
2	PCR to detect *Anaplasma ovis* and *A*. *marginale* in blood samples	2011	Khuzestan	Sheep (n = 109)	[140]
3	PCR to detect *Anaplasma ovis and A*. *marginale* in blood samples	2011	Khuzestan province	Sheep (n = 119)	[141]
4	PCR to detect *Anaplasma ovis*	2012	Tehran	Sheep (n = 20)	[142]
5	PCR to detect *Anaplasma bovis* in salivary glands of ticks	2012	Mazandaran	Ticks (n = 618)	[143]
6	PCR to detect *Anaplasma ovis* in ticks	NS	Fars	Ticks collected from sheep (n = 100)	[144]
7	Nested PCR to detect *Anaplasma phagocytophilum* from cattle’s blood	2007	Isfahan	Cattle (n = 150)	[145]
8	Nested PCR to detect *Anaplasma ovis* and *A*. *bovis* in blood samples	2008	Mazandaran	Hard ticks (n = 101), sheep, cattle, goats (n = 78) and human blood samples (n = 40)	[146]
9	Nested PCR to detect *Anaplasma ovis* and *A*. *marginale* in blood samples	2013	West-Azerbaijan	Blood samples (n = 100) from cattle and sheep	[147]
10	Nested PCR to detect *Anaplasma ovis* in ticks	2013–2014	Sistan and Baluchestan	Ticks (n = 369) from cows, goats and sheep	[56]
11	Nested PCR to detect *Anaplasma ovis* in ticks	NS	Qom	Ticks (n = 278)	[148]
12	Nested PCR to detect *Anaplasma ovis* in ticks	NS	East Azerbaijan, Gilan, South Khorasan, Yazd	Ticks (n = 384)	[149]
13	Microscopic examination for presence of *Anaplasma ovis* in blood smears.	1999–2002	Khorasan Razavi	Cattle (n = 160), Sheep (n = 391) and goats (n = 385)	[150]
14	Microscopy and Nested PCR to detect *Anaplasma bovis* in blood samples	2007	Isfahan	Cattle (n = 150)	[151]
15	Microscopic examination of blood films for *Anaplasma phagocytophilum*	2015	Tehran	Dogs (n = 61)	[152]
C	**Ehrlichiosis**				
1	PCR to detect *Ehrlichia chaffeensis* in *Ixodes ricinus* ticks	2003	Mazandaran	Unfed adult ticks (n = 98)	[153]
2	PCR to detect *E*. *canis* in dogs’ blood samples	2009–2010	Rhazavi Khorasan	Dogs (n = 250)	[154]
3	PCR to detect *E*. *canis* in dogs’ blood samples	2013	Alborz and Tehran	Dogs (n = 240)	[155]
4	PCR to detect *Ehrlichia canis* in blood samples obtained from cattle and stray dogs	2017	Fars	Stray dogs (n = 280)	[156]
5	PCR to detect *Ehrlichia canis* in ticks	NS	Fars	Ticks (n = 89)	[144]
6	PCR to detect *Ehrlichia canis* in blood samples from dogs	NS	Kerman	Dogs (n = 100)	[157]
7	Nested PCR to detect *Ehrlichia canis* in ticks and blood samples from dogs	2011–2012	Ardabil	Dogs (n = 36) and Ticks (n = 146)	[158]
8	Nested PCR to detect *Ehrlichia canis* in ticks	NS	East Azerbaijan, Gilan, South Khorasan, Yazd	Ticks (n = 384)	[149]
9	ELISA test of *Ehrlichia canis* in dogs’ sera	2007–2008	Kerman	Dogs (n = 123)	[159]
10	ELISA test of *E*. *canis* in dogs’ sera	2008–2010	Khuzestan	Companion dogs (n = 198)	[160]
11	Microscopic examination of blood films for *Ehrlichia ewingii*	2015	Tehran	Dogs (n = 61)	[152]
**D**	**Spotted fever group rickettsioses**				
1	IFA and ELISA tests of serum samples from humans and animals	1995	Iran	Human sera (n = 40) and animal sera (n = 40)	[161]

*NS–Not stated*.

Of the 63 eligible studies that were reviewed, 36 (57.1%) reported on coxiellosis (Q fever), 15 (23.8%) on anaplasmosis, 11 (17.5%) on ehrlichiosis and 1 (1.6%) on SFG rickettsioses. Collectively, the different studies were reported in 22 (70.9%) of the 31 provinces of Iran, save for the SFG rickettsioses study which was conducted across four different countries among them Iran. Precisely, coxiellosis studies were reported in 19 provinces, anaplasmosis in 14 and ehrlichiosis in 12 provinces in Iran. However 9 of the 22 provinces had up to three diseases (anaplasmosis, ehrlichiosis and coxiellosis) reported jointly within a single province. In some instances, the coverage area of some studies extended beyond one province to include several provinces.

**Fig 1:**
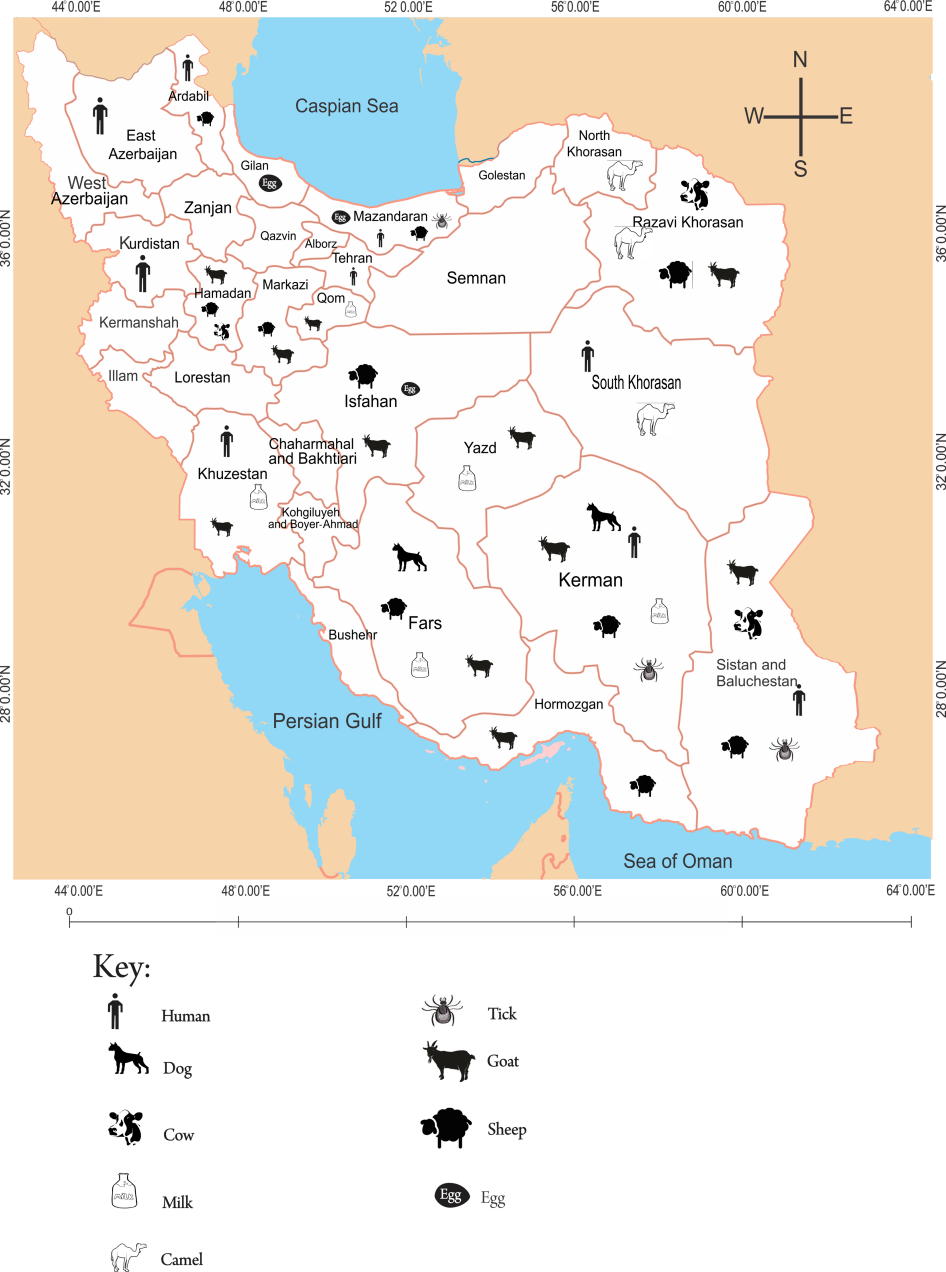
Geographical distribution of domestic animals, animal products, arthropods and human beings from which studies on coxiellosis were conducted in Iran.

**Fig 2:**
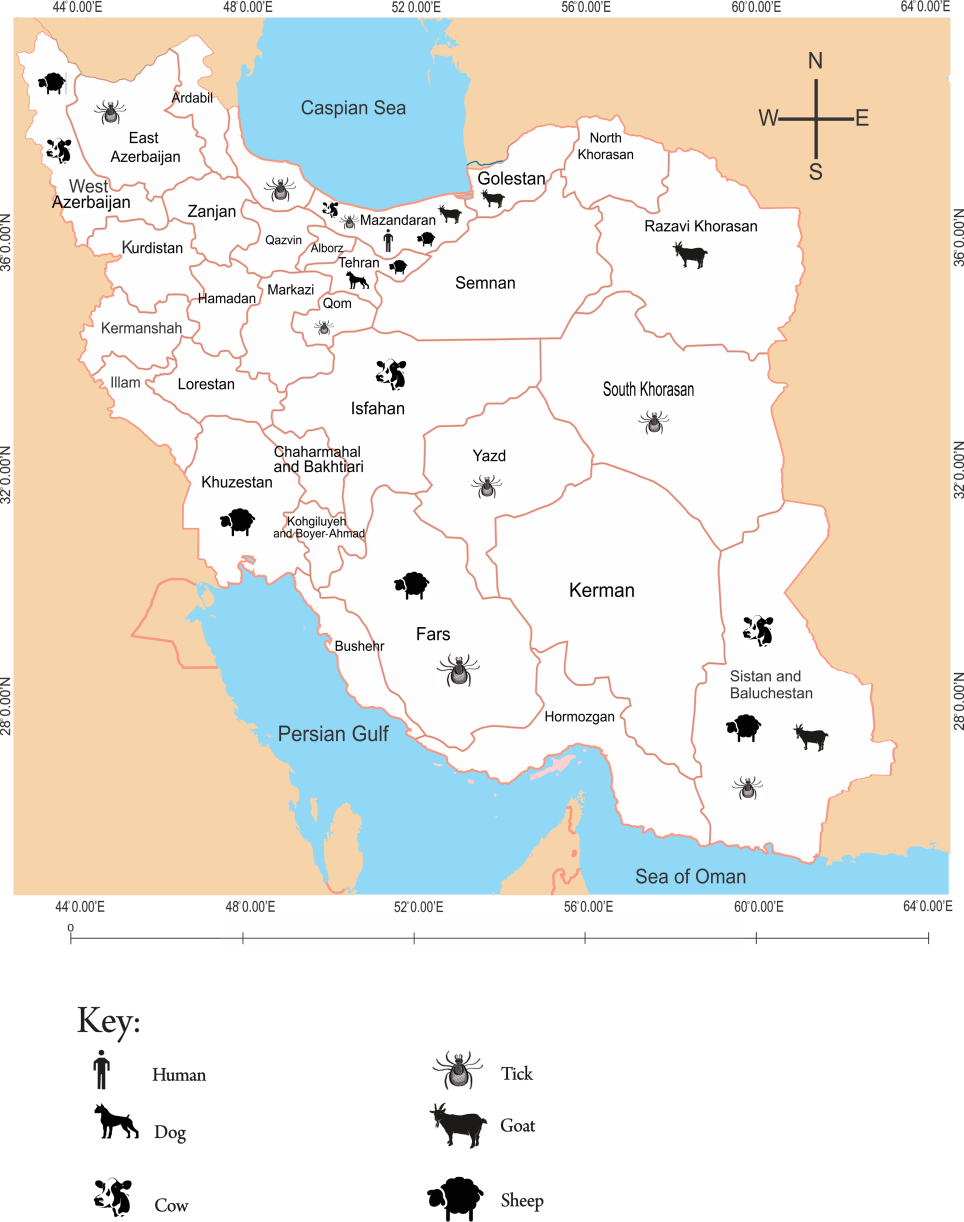
Geographical distribution of domestic animals and ticks from which studies on anaplasmosis were conducted in Iran.

**Fig 3:**
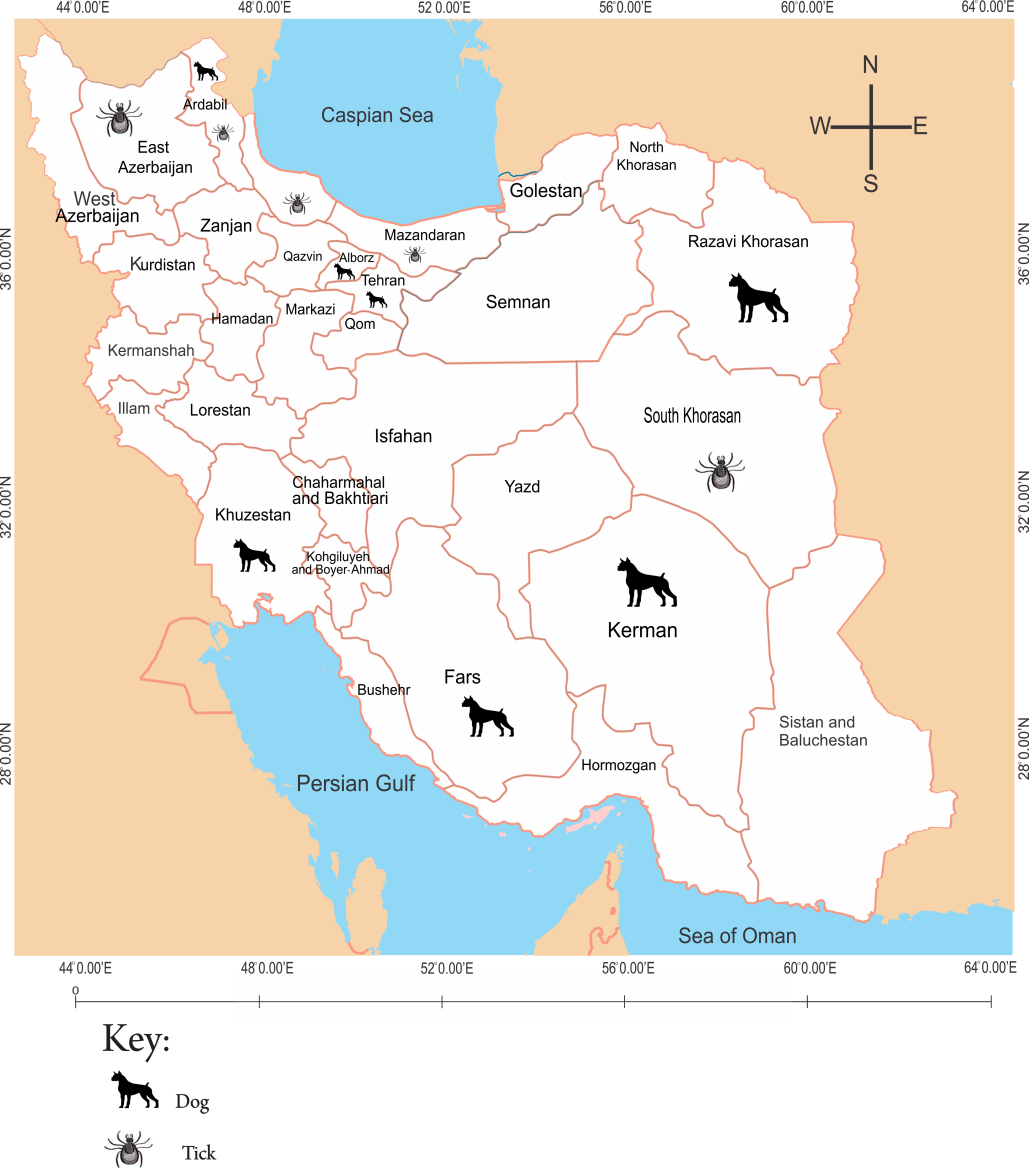
Geographical distribution of dogs and ticks from which studies on ehrlichiosis were conducted in Iran.

Geographically, a large proportion of studies on coxiellosis were distributed in provinces located to the southern (46%) and northern (41.8%) parts of the county (Fig 1), while most studies on anaplasmosis (64.3%) and ehrlichiosis (58.3%) were concentrated to the northern part of the country (Figs 2 and 3). The studied organisms varied among different studies, but were collectively limited to a few domestic animals (cattle, sheep, goats, camels and dogs), animal products (milk and eggs), arthropods (ticks) and human subjects.

As regards diagnostic method used, most (69.4%) studies on coxiellosis utilized serological surveys based on Enzyme Linked Immunosorbent Assay (ELISA), while 25% of the studies utilized molecular surveys based on Polymerase Chain Reaction (PCR). Systematic reviews accounted for 5.6% of all the coxiellosis studies. On the other hand, molecular techniques (PCR) were utilized for most (86.7%) anaplasmosis studies, with the remaining studies (13.3%) being parasitological in nature. As for ehrlichiosis, most (72.7%) studies utilized molecular techniques (PCR), 18.2% utilized serological surveys (ELISA), while 9.1% were parasitological surveys in which microscopy was used. Although majority of the studies presented valid results, few were biased in risk presentation, with some lacking CI, while others had poor precision of CI. The results presented by the various researchers are discussed in details herein.

### Studies on coxiellosis in Iran

Coxiellosis (Q fever) studies were reported in a number of domestic animals, animal products ticks and humans. Geographically, a large proportion of the studies were conducted to the southern (46%) and northern (41.8%) parts of the country, with a small proportion being conducted in the central (4.7%), western (4.7%) and eastern (2.3%) parts of Iran. These studies were collectively reported in 19 (61.3%) of the 31 provinces of Iran. However, the distribution of the studies in the 19 provinces was uneven as a relatively large proportion of the studies were concentrated in 3 of the 19 provinces *i*.*e*. Kerman 11 (25.6%), Sistan and Baluchestan 5 (11.6%) and Mazandaran 4 (9.3%) provinces, leaving the remaining 16 provinces with between 1 and 3 studies; majority (11 out of 16) with just a single study each (Table 1).

Domestic animals (cattle, sheep, goats, camels and dogs), animal products (milk and eggs), arthropods (ticks) and human subjects (febrile patients, pregnant women and individuals with high risk occupations like butchers, slaughter house workers, veterinary officers among others) were the main units of analysis in the various studies that reported on *C*. *burnetii* in Iran. However, majority of the studies reported on small ruminants *i*.*e*. sheep (22.6%) and goats (17.1%), with the dogs, cattle and camels being investigated in 9.4%, 7.5% and 1.9% of the *C*. *burnetii* studies, respectively. *Coxiella burnetii* studies reporting on human subjects accounted for 17.0%, while studies on ticks accounted for 15.1% of all the studies in Iran.

As regards animal products, a total of 5 (9.4%) of all the Q fever studies investigated *C*. *burnetii* antibodies in bulk milk samples collected from commercial dairy cattle and goats, while one study (1.9%) reported on poultry eggs collected from chicken, ducks, goose, quails and ostriches drawn from three provinces (Isfaham, Gilan and Mazandaran) in Iran.

### Prevalence of *C*. *burnetii* infection in domestic animals, animal products and ticks in Iran

Among all the domestic ruminants (cattle, sheep and goats) studied, the herd prevalence was relatively higher than individual prevalence (Table 2). In addition, individual pooled prevalence among domestic ruminants was lowest (0.83–22.3%) in cattle [106, 121, 128] and highest (22.4–65.78%) in goats [106, 114, 117, 121, 138]. Individual pooled prevalence among sheep was intermediate of the two at 19.5–36.5% [110, 112, 117, 121, 126, 137, 162, 163].

**Table 2:** Pooled prevalence of Q fever from a variety of organisms in Iran.

	Species/Organisms		Pooled Prevalence (%)	95% CI range	Reference
**A**	**Domestic animals**				
1	Cattle	Individual	0.83–22.3%	0–27.6	106, 121, 128
		Herd	41.4–78.9%	17.9–97	
2	Sheep	Individual	19.5–36.5%	19.8–42.8	110, 112, 114, 117, 121, 126, 162, 163
		Herd	96.1%	89.1–100	
3	Goats	Individual	22.4–65.78	-	106, 114, 117, 121, 126, 138,
		Herd	93.4%	80.2–100%	
4	Camels		28.3–28.7%	21.5–35.6	119
5	Dogs		7.7–11%	-	123, 137
**B**	**Domestic animals’ products**				
6	Milk		2.0–45.4%	-	109, 133, 134,
7	Eggs		1.5–7.7%	-	132
**C**	**Human beings**				
8	At risk individuals (hunters, butchers, vets, health workers)		19.8–68%	16.4–43.2	111, 118, 125, 127, 138
9	Febrile patients		5.3–35.2%	-	107, 113, 120, 124, 131
10	Pregnant women		29.3%	25–34	122
**D**	**Arthropods**				
11	Ticks		4.8–13.1%	-	17, 130, 136

In regard to cattle, the highest (22.3%) *C*. *burnetii* seroprevalence was reported among 246 dairy cattle drawn from 19 commercial herds in Khorasan Razavi province, northeastern Iran [128], while the lowest (0.83%) was reported among 120 dairy cattle in Hamedan province, western Iran [121]. In goats, the highest (65.78%) and lowest (22.4%) *C*. *burnetii* seroprevalence were both reported in the southeastern part of Iran [106, 114], while in sheep, the highest (33.9%) *C*. *burnetii* seroprevalence was reported among sheep drawn from five counties in the southeastern part of Iran, and the lowest (19.5%) among sheep with a history of abortion drawn from 4 provinces in the northeast, west, southwest and central parts of Iran [117].

Only one study investigated *C*. *burnetii* among one-humped camel population drawn from 11 counties spread across 3 provinces (North, South and Razavi Khorsan) in the northeastern part of Iran. From the study, a *C*. *burnetii* seroprevalence of 28.7% was reported among the camels at the individual animal level [119]. Among dogs, two studies reported on *C*. *burnetii* prevalence in Iran, yielding a pooled prevalence of 7.7–11% with tick infested dogs that had been referred to the veterinary teaching hospital of Shahid Bahonar University of Kerman having a higher seroprevalence (11%) compared to the 7.7% prevalence reported among asymptomatic companion dogs in Fars province, in the southern part of Iran [123, 137].

Among the animal products, three studies investigated *C*. *burnetii* antibodies in bulk milk samples; two from dairy cattle and one from dairy goats. The *C*. *burneti* prevalence rate (2.0%) reported in bulk milk obtained from 89 dairy goat herds drawn from five provinces (Fars, Qom, Kerman, Khuzestan and Yazd provinces) was far much lower than the 45.4% reported in bulk milk samples collected randomly from 44 sufficiently large commercial dairy herds in Kerman province, southeast Iran [109, 133] and the 14% reported in a study of 100 bovine milk samples collected from 5 areas of Qom province [132]. Generally, the pooled prevalence reported on milk samples (2.0–45.4%) was relatively higher than that reported among eggs (1.5–7.7%) in a study involving 369 eggs collected from 130 chicken, 104 ducks, 34 goose, 70 quails and 31 ostriches. Out of the 369 eggs tested, only 2 of 130 (1.5%) chicken eggs and 8 of 104 (7.7%) duck eggs were *C*. *burnetii* positive [132].

A total of four studies were conducted among ticks; yielding a *C*. *burnetii* pooled prevalence of 4.8–13.1%. Three of the 4 studies were conducted in the southeastern part of Iran, precisely in Kerman (1 study) and Sistan and Baluchestan (2 studies) provinces, while the remaining study was conducted in Mazandaran province, northern Iran. All the three studies conducted in the southern part of Iran reported presence of *C*. *burnetii* among the ticks investigated while all the ticks investigated in the northern part of Iran tested negative for *Coxiella burnetii*. The investigated ticks were collected from domestic animal’s bodies (goats, sheep and cattle) and others from shrubbery. Collectively, the investigated ticks belonged to nine different species, namely: *Rhipicephalus sanguineus* sensu lato, *R*. *turanicus*, *R*. *bursa*, *Hyalomma anatolicum*, *H*. *excavatum*, *H*. *asiaticum*, *H*. *marginatum*, *H*. *dromedarii*, *H*. *detritum*. Of the nine species, *H*. *anatolicum*, *H*. *excavatum*, and *R*. *sanguineus* sensu lato tested positive for *C*. *burnetii* in nested trans-PCR assay [130, 136, 137, 153].

### Prevalence of *C*. *burnetii* infection among humans in Iran

Almost half (45.4%) the studies on coxiellosis among human subjects were conducted in the southeastern part of Iran, particularly in Kerman and Sistan & Baluchestan provinces, while 27.3% were conducted in the northwestern part of the country. The remaining 18.2% and 9.1% were carried out in the northern and eastern parts of Iran, respectively (Table 2). Among human subjects, *C*. *burnetii* seroprevalence levels varied between febrile patients, pregnant women and individuals with risky occupations (hunters, butchers, health workers, veterinary workers and veterinary students). Most (63.6%) of the coxiellosis studies on human subjects were conducted among febrile patients [107, 113, 120, 124, 127, 129, 131], while 27.3% were conducted among at-risk individuals [111, 115, 116, 118, 125, 138] and 9.1% among pregnant women [122]. Pooled prevalence among at-risk individuals was relatively higher (19.8–68%), than the pooled prevalence among febrile patients (5.3–35.2%). Compared to the other categories of human subjects, a relatively low (29.3%) *C*. *burnetii* seroprevalence was reported among pregnant women in a single study conducted on 400 random samples collected from pregnant women who had been referred to diagnostic laboratories of Ahvaz and Parsabad in the southwest and northern part of Iran, respectively [122].

### Epidemiology of anaplasmosis in Iran

A total of 15 studies reported on anaplasmosis among domestic ruminants (cattle, sheep and goats), arthropods (ticks) and human beings in Iran. Geographically, most (68.4%) of the studies were conducted in the northern part of Iran. Precisely, 5 (26.3%) studies were carried out to the northwest, 9 (31.6%) to the north, 2 (10.5%) to the northeast, 3 (15.8%) in central, 2 (10.5%) to the southeast and 1 (5.3%) to the eastern part of the country. Considering their distribution per province, studies on anaplasmosis were collectively conducted in 14 (45.2%) of the 31 provinces in Iran. More than half (9 out of 14) the provinces where the studies were conducted are located to the northern part of the country. Studies on anaplasmosis were more-less evenly distributed in the 14 provinces with 5 (35.7%) provinces having two reported studies each, while the remaining 9 (64.3%) provinces had a single study focusing on anaplasmosis each.

### Prevalence of *Anaplasma* spp. infection in domestic ruminants, ticks and humans in Iran

A total of five species in the genus *Anaplasma* were isolated from samples obtained from cattle, sheep, goats, ticks and human subjects that were investigated. The five *Anaplasma* species included *Anaplasma ovis*, *A*. *bovis*, *A*. *marginale*, *A*. *centrale* and *A*. *phagocytophilum* (Table 3). Of the five species, *Anaplasma ovis* was the most dominant and was detected in all the study organisms (cattle, sheep, goats, ticks and human beings); though to varying levels. The pooled prevalence of *Anaplasma ovis* was highest among sheep (5.0–87.4%), followed by goats (22.3–63.7%), ticks (4.7–55.5%) and cattle (0.0–22.2%) in that order [139, 140, 141, 142, 143, 144, 145, 146, 147, 148, 151]. *A*. *ovis* was also reported among human beings in a study conducted in Mazandaran province involving 40 human blood samples of which 25% tested positive [146].

**Table 3:** Pooled prevalence of anaplasmosis and ehrlichiosis from a variety of organisms in Iran.

**A**	**Anaplasmosis**			
	**Species/Organisms**	**Prevalence (%) range**	**95% CI range**	**Author**
1	Cattle	*A*. *ovis*: 0–22.2%	-	145, 146, 147, 150, 151
		*A*. *marginale*: 0–19.37%	-	
		*A*. *phagocytophilum*: 1.33%	-	
		*A*. *bovis*: *2*.*66%*	-	
2	Sheep	*A*. *ovis*: 5.0–87.4	-	140, 141, 142, 146, 147, 150
		*A*. *marginale*: 5–53.2%	-	
		*A*. *central*: *3*.*57%*	-	
3	Goats	*A*. *ovis*: 22.3–63.7%	-	139, 146, 150
4	Human being	*A*. *ovis*: 25%	-	146
5	Ticks	*A*. *phagocytophilum*: 5.1%	-	56, 143, 146, 153
		*A*. *ovis*: 4.7–55.5%	-	
		*A*. *bovis*: 2–59%	-	
**B**	**Ehrlichiosis**			
	**Species/Organisms**	**Prevalence (%) range**	**95% CI range**	**Author**
1	Dogs	*E*. *canis*: 0.8–16.6%	-	156, 157, 158,159, 161, 150, 152
		*E*. *ewingii*: 18%	-	
2	Ticks	*E*. *canis*: 1.8–43.8%	-	128, 158

*Anaplasma marginale* was only reported among sheep and cattle with a pooled prevalence of 0–19.37% in cattle and a prevalence of 5% in sheep [132], while *A centrale* was only reported among sheep in a study conducted in Mazandaran province, in which 1 of 28 (3.57%) sheep tested positive. *Anaplasma bovis* was reported among cattle (2.66%) in the central part of Iran [151] and in ticks (59%) collected from goats and sheep in Mazandaran province, northern Iran [143]. *Anaplasma phagocytophilum* was reported among cattle and ticks, with a higher prevalence being reported among ticks (5.1%) than cattle (1.33%) [144, 151].

### Epidemiology of ehrlichiosis in Iran

A total of 11 studies reported on *Ehrlichia* spp. among dogs, cattle and ticks extracted from bodies of domestic animals (cattle and goats) in Iran. The studies were collectively conducted in 12 (38.7%) of the country’s 31 provinces. Over half (7 out of 12, 58.3%) the provinces were concentrated to the northern part of the country, three (25%) to the southern part, one to the Eastern part and one in central Iran. Relatively more studies reported on Ehrlichiosis among dogs (58.3%) than ticks (41.7%) (Table 3).

### Prevalence of *Ehrlichia* spp. infection in domestic animals, ticks and human beings in Iran

In total, three *Ehrlichia* species were reported in the reviewed studies. The most dominant species; *E*. *canis* was reported in 9 (81.8%) of the 11 studies while *E*. *chaffeensis* and *E*. *ewingii* accounted for one study each. *E*. *canis* was isolated from both dogs [153, 156, 157, 158, 159, 160] and ticks [128, 149, 158] while *E*. *chaffeensis* was reported among unfed adult *Ixodes ricinus* ticks in Mazandaran province [153]. *Ehrlichia ewingii* was reported in dogs’ blood films previously collected at random from different veterinary hospitals in Tehran, the capital of Iran [152].

The pooled prevalence of *E*. *canis* was higher among ticks (1.8–43.8%) compared to dogs (0.8–16.6%). In addition, *E*. *ewingii* prevalence of 18% reported among dogs’ blood films was relatively higher compared to the *E*. *chaffeensis* prevalence of 5.1% reported among *Ixodes ricinus* ticks in Ghaemshahr city in Northern Iran (Table 3).

### Spotted fever group rickettsioses

In the current review, only one article reported on spotted-fever group rickettsioses in a study involving 40 samples of human sera collected from 4 countries among them Iran and examined for presence of antibodies to spotted fever group rickettsiae among other pathogens by Kovacova et al. [161]. Of the 40 human sera samples tested, 45% were positive for SFG rickettsiae by ELISA test and 27.5% by IFA test (27.5%).

## Discussion

This review attests to the existence of Coxiellosis, Ehrlichiosis, Anaplasmosis and SFG rickettsioses in different organisms in Iran, pointing to a possible circulation of pathogens that could be maintaining active foci of these diseases in the environment. Coxiellosis, ehrlichiosis, anaplasmosis and SFG rickettsioses being zoonoses, presence of their causative agents in the environment increases the risk of animal-human transmission. From the 63 articles reviewed, most (57.1%) reported on coxiellosis, while anaplasmosis, ehrlichiosis and SFG rickettsioses accounted for 23.8%, 17.5% and 1.6%, respectively.

The presence of various pathogens was confirmed by molecular, serological and microscopic techniques conducted on specimens obtained from humans, ticks, domestic animals and animal products (milk and eggs) drawn from a number of provinces in Iran. A large proportion (66%) of Q fever seroprevalence studies used ELISA technique to detect *Coxiella burnetii* antibodies, with only 25.7% using PCR techniques. On the contrary, most studies on anaplasmosis (86.7%) and ehrlichiosis (72.7%) utilized molecular techniques for pathogen detection. Although ELISA technique has been around over the last 40 years, its’ sensitivity is often considered as being low since it is only designed for clinical diagnosis of acute infections with high antibody titres, and therefore those with lower antibody titres may be easily missed. In addition, ELISA technique involves a number of variables, such as reagent selection, temperature, volume measurement, and time, which if not correctly adjusted can affect subsequent steps and thus influence the test outcome leading to unreliable results. This makes PCR techniques relatively accurate and therefore more reliable [112, 163]. Besides, PCR based diagnostic assays that have been developed to detect *C*. *burnetii* DNA in cell cultures and clinical samples are most ideal compared to conventional methods; most of which are dangerous and time consuming [164]. In addition, PCR techniques are sensitive and specific for confirming diagnosis at the onset of acute clinical signs when antibody tests are usually still negative. The use of molecular techniques in future surveillance of tickborne zoonoses in Iran will therefore provide more accurate and reliable epidemiological data.

### Prevalence of coxiellosis in Iran

Geographically, over half (57.1%) the studies in this review reported on coxiellosis in 19 of the 31 provinces in Iran. However, the distribution of these studies was not uniform as most studies were concentrated in two provinces (Kerman and Sistan & Baluchestan), both of which are located to the southeastern part of Iran. This skewed distribution excludes 12 provinces which collectively cover a substantial portion of the country. The central, eastern and western parts of Iran were particularly excluded, implying that the findings emanating from the reviewed articles may not reflect the actual epidemiology and prevalence of Q fever in Iran.

The ability of Q fever pathogenic agents (*Coxiella burnetii*) to be transmitted from animal reservoirs to humans by inhalation of infected aerosols makes coxiellosis easily transmissible among different organisms over large geographical areas [165]. Indeed, airborne transmission of *C*. *burnetii* is a well-documented phenomenon in many regions across the world [166, 167]. Likewise, livestock movements across regional and national boundaries may also contribute to the spread of coxiellosis from one region to another [168]. Therefore, while studies on coxiellosis may not have covered the entire country, it is highly likely that *C*. *burnetii* pathogens exist throughout the country owing to the free movement of animals and people from one region to another. This calls for more studies involving a wider range of domestic and wild animals throughout the entire country to ascertain the actual *C*. *burnetii* prevalence in Iran.

### Prevalence of C. *burnetii* infection among domestic animals

*Coxiella burnetii* bacterium has a wide range of hosts including wild and domestic mammals, birds, reptiles and arthropods [169]. However, domestic ruminants (primarily cattle, sheep and goats) are the most important and frequent source of human infection of *C*. *burnetii* [170], although transmission from dogs and cats has been documented as well [171].

Once shed, the *C*. *burnetii* bacterium may remain infective in the environment for several months [172], during which time the bacterium survives in arthropod hosts such as ticks from which they spread into ruminants. Based on findings emanating from this review, a majority of *Coxiella burnetii* studies were conducted on small ruminants *i*.*e*. sheep (22.6%) and goats (17.1%). Studies on *C*. *burnetii* antibodies in goats were conducted in 8 provinces in Iran, with the highest (65.78%) seroprevalence recorded in Kerman province and the lowest (0%) in Markazi province. Similar studies conducted among goats elsewhere yielded varied *C*. *burnetii* seroprevalence levels; most of which were lower than those reported in Iran. For instance, *C*. *burnetii* pooled prevalence of 20–46% was reported in Kenya [173] and 0.8–60.6% in China [174]. In addition, *C*. *burnetii* prevalence of 7.8%, 13.7%, 9.52%, 8.8%, 6.5%, 8.7% and 13% were reported in Netherland, Bulgaria, Bangladesh, Albania, Northern Greece, Spain and Italy, respectively [175, 176, 177, 178, 179, 180].

Among sheep, *C*. *burnetii* prevalence studies documented in this review were conducted in 10 provinces in Iran, with a herd prevalence of 96.1% and an individual pooled prevalence of 19.5–36.5% being reported. In comparison to the prevalence levels reported among sheep in Iran, relatively lower *C*. *burnetii* prevalence rates have been reported in different countries. For instance, a *C*. *burnetii* prevalence of 21% was reported in Spain [175], 13.5% and 20% in Turkey [181, 182], 18.9% in Cyprus [183], 10% in USA [184], 1.3% in Germany [185], 2.4% in Netherlands [179], 5% in China [174], 11.6% in Bulgaria [180], and a pooled prevalence of 11–33% in Africa [19].

The relatively high *C*. *burnetii* seroprevalence reported among smaller ruminants in Iran as compared to other animals makes them perfect reservoirs and potential agents of *C*. *burnetii* transmission to other animals and humans. According to Maurin and Raoult [172], massive shedding of *C*. *burnetii* during abortions makes sheep and goats the main reservoirs responsible for human infection. Q fever outbreaks resulting from infected ruminants are not new as they have been reported in many parts of the world in the past. Analysis of human Q fever outbreaks in Europe confirmed that the outbreaks were associated with small ruminants and not cattle [186], with human infections being attributed to inhalation of contaminated aerosols [187].

Between 2012 and 2014, a Q fever outbreak reported in Australia was attributed to a nearby intensive goat and sheep farming venture with a *C*. *burnetii* prevalence of 15% being reported among goats in the farm at the time [188]. Likewise, between 2004 and 2009, a number of human Q fever outbreaks were reported in Bulgaria, Croatia, France, Germany and Italy; all of which were attributed to sheep farming [189]. These findings attest to the significant role that small ruminants (mainly goats and sheep) play in Q fever transmission to humans. Given the high *C*. *burnetii* seroprevalence rates reported among goats and sheep in Iran, the possibility for a human outbreak cannot be ruled out; though more studies are required to ascertain such a possibility in future.

Among cattle, *C*. *burnetii* prevalence was reported in three provinces in Iran, with the highest prevalence reported in Sistan and Baluchestan province and the lowest in Hamedan province. Following this review, the pooled prevalence reported among individual cattle was 0.83–22.3%, while the herd prevalence was 41.1–78.9%. These rates were relatively higher compared to the 6.2% *C*. *burnetii* seroprevalence rates reported among cattle in Northern Ireland [190], 8.5% in Bulgaria [180], 15% in China [174], 16.0% in Netherlands [191], 7.8% in Germany [185], 14.3% in the Central African Republic [192] and 14.5% in Mexico [138]. However, the rates reported among cattle in Cyprus (22.7%) and Cameroon (30.4%) [193] were higher than those reported in Iran. The varying rate of *C*. *burnetii* prevalence among different domestic ruminants in different regions is consistent with findings of high regional variability reported among farm animals in four European countries [194]. The regional variations could be attributed to some confounding factors that may be in operation at the local scale which need to be investigated further.

In the current review, only a single study reported on *C*. *burnetii* seroprevalence among one-humped camels drawn from North Khorsan, South Khorsan and Razavi Khorsan provinces, in the northeastern part of the country [119]. The *C*. *burnetii* prevalence of 28.7% reported among camels in Iran was however lower compared to 51.6% reported among camels in Saudi Arabia and 80% reported in Chad [195, 196]. The Food and Agriculture Organization [197] estimates that nearly 150,000 dromedary camels live in desert areas (South and Central) of Iran, most of which are scattered across 19 of the country’s 31 provinces. The country’s camels account for about 0.56% of the world’s camel population and 3.8% of the Asian camel population [197]. Despite the large camel population in Iran, only a single study involving 167 camels was conducted in the northern part of Iran. This is seen as insignificant and cannot be relied upon to give a general prevalence of *C*. *burnetii* among camels in Iran. Besides, not a single study of *C*. *burnetii* prevalence among camels was conducted in the desert areas of the country that lie to the south and central part of the country, where the largest population of camels are found. Given the importance of camels to pastoralists; more so in arid regions of Iran, more studies are needed to establish the true prevalence of *C*. *burnetii* among camels in the country.

Dogs are well-described reservoirs for *C*. *burnetii* [198]. In the current review, only two studies investigated *C*. *burnetii* prevalence in Iran; one among asymptomatic companion dogs in Fars province and the other among dogs taken to a veterinary hospital in Kerman province. From the two studies, a pooled prevalence of 7.7–11% was reported [123, 137]. Though Q fever is less prevalent in dogs compared to domestic ruminants, dogs that are exposed to infected wildlife carcasses, sick farm animals and their offspring or livestock environment where the *C*. *burnetii* bacterium is present are at higher risk; with the most common mode of transmission being ingestion or inhalation of contaminated aerosols [199, 200]. Presence of *C*. *burnetii* antibodies in asymptomatic companion dogs is of particular importance as they can transmit the disease to humans; thus posing a potential risk of Q fever outbreak [201]. However, the role of dogs in the transmission of *C*. *burnetii* to humans remains uncertain in Iran, which necessitates extensive seroprevalence studies of dogs across the country.

### Prevalence of *C*. *burnetii* infection in ticks

Over 40 tick species are known to harbor *C*. *burnetii* bacterium; thus serving as indicators of its circulation in nature [202]. Ticks play a critical role in the transmission of *C*. *burnetii* particularly among wild vertebrates [203], though direct transmission of this agent to humans from infected ticks is still controversial and not properly documented [204, 205]. In the current review, four studies investigated *C*. *burnetii* prevalence in ticks collected from animal bodies and shrubbery. *Coxiella burnetii* bacteria were detected in three (all located to the southeastern part of the country) of the four studies, with a pooled prevalence 4.8–13.1% being reported. The overall prevalence of *C*. *burnetii* in ticks from Iran was relatively higher than the 0.1% reported in Spain [206], 0% in Europe and Germany [207]; 2.5% in Slovakia and Hungary [208], 0.8% in Greece [209] and 2% in Egypt [210].

Tick species are probably the reason for the observed differences in *C*. *burnetii* from region to region. In the current review, nine tick species belonging to two genera (*Rhipicephalus* and *Hyalomma*) were extracted from animal bodies and shrubbery in Iran. Out of these, 3 species: *Hyalomma anatolicum*, *H*. *excavatum*, and *Rhipicephalus sanguineus* tested positive for *C*. *burnetii*. The detection of *C*. *burnetii* in 3 tick species is however not surprising since over 40 species have been found to harbor *C*. *burnetii* bacterium in many parts of the world [208].

### *Coxiella burnetii* prevalence in animal products

In the current review, only four studies reported on *Coxiella burnetii* prevalence in animal products; three on bulk milk samples (2 from dairy cattle and one from goats) and one on poultry eggs collected from chicken, goose, quail, ostrich and ducks. The pooled prevalence of *C*. *burnetii* was higher in milk (2.0–45%) than eggs (1.5–7.7%). In addition, *C*. *burnetii* prevalence in bulk milk samples from dairy cattle (14–45%) was higher that bulk milk samples from goats (2%). The current findings are consistent with those of Eldin et al. [211] whose study also reported the prevalence of *C*. *burnetii* in dairy products to be significantly higher than products from goats or ewes in France. The *C*. *burnetii* prevalence rates reported in bulk milk samples in Iran were however higher than those reported among bovine milk in Switzerland (4.7%) [212], but lower than the 90% reported among dairy herds in the USA on the basis of bulk tank milk testing over a three-year period [213].

According to Cerf and Condron [214], *C*. *burnetii* pathogens can be resistant to physical and chemical factors such as heat, dryness and most disinfectants. This makes it possible for the pathogen to survive for days and weeks in animal products such as milk, cream, butter and cheese, posing a risk of pathogen transmission to humans through consumption of raw animal products like milk and eggs [215]. Most often than not, infected animals shed *C*. *burnetii* into the environment through milk, colostrum, eggs, urine, vaginal discharges; especially in birth products [14]. Cases of human Q fever outbreaks associated with consumption of dairy products have been reported in many parts of the world. For instance, Fishbein and Raoult [216] reported a Q fever outbreak in a psychiatric institution in southern France where seropositivity rates for *C*. *burnetii* were significantly higher among patients who consumed unpasteurized milk products. Another study conducted in Japan detected *Coxiella* DNA in commercial chicken eggs and mayonnaise using nested-PCR targeting *Coxiella* outer membrane protein gene (*com1*) [217].

Raw milk consumption was identified as a risk factor for *C*. *burnetii* seropositivity among dairy cattle farmers in Germany [218]. While *C*. *burnetii* prevalence levels reported among animal products in Iran were relatively higher compared to those reported in other studies elsewhere, the number of studies (4) conducted in Iran were however insufficient to give an accurate overall prevalence of *C*. *burnetii* among animal products. This calls for additional studies focusing not only on eggs and milk but also on many other animal products such as cheese, yoghurt and butter. In addition, the studies should also be conducted on milk from other animals such as camels, ewes and buffaloes since they also play an important role in the economy of the country.

### *Coxiella burnetii* prevalence among human subjects

In the current review, 12 studies focusing on *C*. *burnetii* prevalence among human subjects were collectively reported in 12 (38.7%) of the 31 provinces in Iran. However most (45%) of the studies were concentrated to the southeastern part of Iran, especially in 2 provinces (Kerman and Sistan and Baluchestan), thus excluding a large portion of the country. Nevertheless, presence of *C*. *burnetii* pathogens in a few provinces in Iran could signal a countrywide distribution given the ability of *C*. *burnetii* infected aerosols to be transported by wind over long distances coupled with the bacterium’s ability to survive in the environment for long periods of time. Tissot-Dupont [166] concurs and opines that some Q fever outbreaks are related directly to the speed and frequency of wind.

In the current review, Q fever seroepidemiological studies on human subjects were generally categorized into three; though disproportionately. The categories included: pregnant women with only 1 of 12 (8.3%) studies, individuals with high risk occupations with 5 (41.7%) studies and febrile patients with a total of 6 (50%) studies. *Coxiella burnetii* seroprevalence rates varied among the different categories of study subjects; with individuals considered at risk based on their occupation recording the highest seroprevalence (19.8–68%) followed by febrile patients (5.3–35.2%). The single study conducted among 400 pregnant women reported a 29.3% *C*. *burnetii* seroprevalence.

As is evident from the findings of this review, Q fever is primarily an occupational hazard with those in close contact with domestic animals and animal products like farmers, veterinarians, slaughterhouse workers, laboratory personnel, health care workers being at a relatively higher risk [167]. According to Parker et al. [219] *C*. *burnetii* transmission in humans is dependent on a number of factors among them the nature of work or occupation, frequency of contact with live infected animals, frequency of contact with carcasses and tissues of slaughtered animals, proper use of personal and environmental protective gears as well as individual attitude and level of knowledge among those at risk.

Another common cause of human infection with *C*. *burnetii* is inhalation of infectious aerosol or contaminated dust containing air-borne bacterium, which is regarded as the major route through which human beings acquire the disease. A single inhaled *C*. *burnetii* bacterium has the capacity to produce clinical illness [220]. Other transmission routes of Q fever in human that have been identified include consumption of contaminated animal products, skin or mucosal contact, tick bites, blood transfusion, sexual transmission and embryo transfer [167, 219]. Researchers acknowledge that Q fever is common among workers in livestock and animal products trade especially those dealing with cattle, sheep and goats [221].

Among febrile patients (majority with brucellosis like symptoms), *C*. *burnetii* pooled prevalence of 5.3–35.2% was reported, with the highest prevalence being among patients admitted to Boo-Ali Hospital in Zahedan County in Sistan and Baluchestan province, southeast Iran [113]. This prevalence rate was relatively higher compared to the 3.85% reported among febrile patients in Mali [221], 5.8% in Croatia [222], 2.29% in Denmark [223] and 2.07% in France [224]. A study performed on British soldiers with fever of unknown origin in Afghanistan established that 26% of the soldiers had acute Q fever [225]. From the foregoing, it is evident that the prevalence of coxiellosis among febrile patients suspected of having brucellosis was high. The major cause of infections reported in a number of provinces in Iran could be contact with infected livestock and contaminated dairy products. Therefore, necessary health measures for disease prevention targeting the whole country are required.

Pregnant women are also at a higher risk of Q fever infection which is potentially dangerous to them as it may cause serious complications for both the foetus and the mother; especially if it occurs in the early stages of pregnancy [226]. In the current review, only one study was conducted among 400 pregnant women drawn from two provinces in the northern and southwestern parts of Iran, from which a *C*. *burnetii* seroprevalence of 29.3% was reported [88]. This prevalence was, however, much higher compared to the rates reported among pregnant women in other countries such as France (2.6%), Canada (4%), London (4.6%), Bulgaria (7.7%) and Netherlands (9.1%) [227, 228, 229, 230]. While only one study reported on *C*. *burnetii* seroprevalence among pregnant women in Iran, the prevalence reported was considerably high to be ignored. Infection with coxiellosis is a major cause for concern especially for pregnant mothers and their unborn babies given the adverse health effects that they are bound to trigger such as spontaneous abortion, intrauterine growth retardation, intrauterine fetal death and premature delivery [231].

This systematic review established that the human Q fever studies in Iran were restricted to only three categories of human subjects (*i*.*e*. febrile patients, pregnant mothers and people with risky occupations), thus excluding the general public who form a majority of the country’s population. As it is therefore, these findings cannot be generalized to reflect the actual prevalence of coxiellosis in Iran. More studies on Coxiellosis among human subjects are thus needed to ascertain the actual prevalence of the disease in the general population. Future studies should broaden their scope to include both at risk individuals and the general public who may not necessarily be regarded as being at risk. In addition, coverage should extend to all provinces within Iran.

Generally, despite surmounting evidence from epidemiological studies of the prevalence of *C*. *burnetii* pathogens in Iran, only seven species of domestic animals, different tick species, two animal products (milk and eggs) and human subjects have been investigated for prevalence of *C*. *brunetii* in the country. The limited number of organisms and animal products excludes many other animals such as cats, pigs, horses, birds, rabbits, fish, rodents, a number of mammals and arthropods that have been reported elsewhere as not only harboring *C*. *burnetii* pathogens but also playing a role in transmission [129].

In addition, many studies have also highlighted the presence of *C*. *burnetii* in a number of wild animals in other parts of the world. For instance, Webster *et al*. [232] detected antibodies to *C*. *burnetii* in wild brown rats on farms in the United Kingdom; while Madariaga [233] reported a seroprevalence of between 7 and 53% among brown rats in Oxfordshire, UK. *Coxiella burnetii* antibodies have also been isolated from hares and wild rabbits [234], coyotes, skunks, foxes, deer, wood rats, squirrels, bush rabbits, wild mice and buffaloes in different parts of the world [235]. While the role of wild animals as reservoirs of *C*. *burnetii* is well documented elsewhere, not a single study focused on wildlife in Iran. On the whole, active surveillance and more research studies targeting a broad range of organisms across all provinces of Iran are necessary for successful preventative planning and control of *C*. *burnetii* infections in the country.

### Prevalence of anaplasmosis in Iran

In the current review, 15 studies reported on anaplasmosis in 14 (54.2%) of the 31 provinces in Iran. Most of the provinces were located to the northern part of the country [139, 141, 144, 146, 147, 150]. This however excludes more than half (54.8%) the provinces in the country, leaving a large portion of Iran out. Besides, only 1–2 studies were conducted in each of the 14 provinces, making the study findings too limited to give a reflection of the actual prevalence of anaplasmosis in Iran.

Collectively, domestic ruminants (cattle, sheep and goats), ticks and human beings were investigated in the 15 studies, though disproportionately. Of the 15 studies, 36% were conducted on sheep, 24% on cattle, 20% on ticks, 16% on goats and 4% on human subjects. From these, five species in the genus *Anaplasma* were detected, namely: *Anaplasma ovis*, *A*. *bovis*, *A*. *marginale*, *A*. *centrale* and *A*. *phagocytophilum*. Only two (*A*. *platys* and *A*. *carpa*) of the seven recognized *Anaplasma* species worldwide were not reported in any of the 15 studies in Iran.

In the current review, *A*. *ovis* was the most dominant pathogen having been detected collectively in all categories (human beings, domestic ruminants and ticks) of studied organisms. Pooled prevalence of *A*. *ovis* was highest (5.0–87.4%) among sheep, followed by goats (22.3–63.7%), ticks (4.7–55.5%), human (25%) and cattle (2.66–22.2%). These findings point to the greater role that small ruminants (sheep and goats) play as reservoirs of *A*. *ovis*. First described in sheep in 1912, *Anaplasma ovis* is widely distributed in Asia, Africa, Europe and USA [236, 237], and now infects goats, cattle and some wild ruminants [238]. Moreover, the DNA of *A*. *ovis* has been detected in milk samples from goats and sheep in China [239]. Apart from domestic ruminants and animal products, *A*. *ovis* has also been detected in different domestic and wild animals with varying prevalence rates among them mongolian gazelle (52.2%) [238], dogs (6.1%) [240], red deer (32.0%) and sika deer (20.0%) in China [238].

In the current review, *A*. *ovis* was also detected in 25% of the 40 human blood samples examined in a study conducted within Mazandaran province, northern Iran. This corroborates the *A*. *ovis* variant that was also detected in a patient in Cyprus, indicating the zoonotic potential of the pathogen [241]. Other *Anaplasma* species were also detected in Iran though to a lesser extent and on a limited number of organisms. For instance, *A*. *marginale* was reported among sheep and cattle with a relatively higher prevalence reported among cattle compared to sheep [132]. Studies concur that *A*. *marginale* occurs mostly in cattle, but has also been detected in a number of wild animals including deer, bighorn sheep, black wildebeests, pronghorn antelopes, elks, giraffes and bison in other studies conducted elsewhere [242]. *A*. *marginale* has also been detected in water buffaloes in Brazil [242]. Unfortunately, despite clear evidence of *A*. *marginale* prevalence in many wild animals in other parts of the world, not a single study reported on *A*. *marginale* among wild animals in Iran; leaving a large knowledge gap that needs to be filled.

In the current review, *A*. *centrale* was only reported among sheep drawn from Mazandaran province, in which only 1 out of 28 sheep tested positive. *Anaplasma centrale* is considered less pathogenic, whose infection causes only mild effects [34]. The pathogen has even been used as a live vaccine for cattle in Israel, South Africa, South America and Australia [243]. The less pathogenic nature of *A*. *centrale* may not have aroused the interest of researchers to this particular pathogen in Iran. Nevertheless, more studies are still required to establish the prevalence of *A*. *centrale* among different organisms in the country.

In the current review, *Anaplasma bovis* prevalence of 2.66% was reported among cattle in the central part of Iran [151] and 59% among ticks extracted from goats and sheep bodies in Mazandaran province, northern Iran [143]. While the major reservoirs of the *A*. *bovis* pathogen are known to be cattle and goats [244], the prevalence rate reported among ticks in the current review was 22 times higher than that reported among cattle. According to Donatien and Lestoquard [245], *A*. *bovis* was first discovered in cattle but has since been detected in many domestic and wild animals in many countries around the world among them Italy, Brazil, South Africa, Korea, China, Tunisia, Spain, Japan and United States of America.

Among the reviewed articles, *A*. *phagocytophilum* was detected in 5.1% of the ticks and 1.33% of the cattle that were investigated. The rate of *A*. *phagocytophilum* infectivity reported among *Ixodes ricinus* ticks in northern Iran by Bashiribod *et al*. [153], and those reported in a study in Austria (5.1%) by Sixl *et al*. [246] were comparable to the rates reported in the current review, but slightly higher than the rates reported in northwest Poland (4.5%) [247], and Germany (4.1%) [248]. Recent investigations show that many species of ticks except *I*. *persulcatus* could carry *A*. *phagocytophilum* pathogen [249].

Nevertheless, presence of *A*. *phagocytophilum* in cattle and hard ticks in Iran is of importance as it portends a possible risk of transmission to humans in different parts of the country. Cross-border movements of persistently infected organisms may contribute to the spread of variants between different geographical areas [250]. Smaller ruminants (goats and sheep) are also prone to infection by *A*. *phagocytophilum*. Besides, there is evidence that sheep are natural reservoir hosts for *A*. *phagocytophilum* in the United Kingdom [251], while other studies suggest that *A*. *phagocytophilum* pathogen is normally persistent in sheep [250]. The pathogen has also been detected in many other domestic animals like horses in a number of countries especially in Great Britain, Denmark, Sweden, Switzerland, France, Germany, Czech Republic and Italy [252], as well as among dogs, cats and Ilamas [253].

Cases of *A*. *phagocytophilum* infection among wild animals have also been reported in different parts of the world. In the USA and Europe for instance, wild rodents such as the white-footed mice [254] and white-tailed deer are well known as natural reservoirs for *A*. *phagocytophilum* [255]. Another study in Slovenia revealed that red and roe deer were infected with *A*. *phagocytophilum* in about 86% of the cases [256]. *A*. *phagocytophilum* strains have also been identified as being potentially virulent to the roe and red deer in Northeast Poland and Slovenia as well as in wild ruminants including Cervidae [257]. Migrating birds are thought to be important dispersal agents of *A*. *phagocytophilum* infected *I*. *ricinus* in Europe [258]. Despite overwhelming evidence of *A*. *phagocytophilum* among wild animals including birds, not a single study focused on wild animals in Iran. Although the reviewed studies on *A*. *phagocytophilum* did not focus on human subjects in Iran, serological evidence of human infection with *A*. *phagocytophilum* exists in Korea and other parts Asia [259]. Therefore, while conducting studies among domestic and wild animals, attention should also be given to human subjects across the county.

The current review confirms the prevalence of *Anaplasma* spp. infection in parts of Iran as well as the potential role that domestic ruminants and ticks could be playing in the transmission of the pathogens across the country. However, the number of studies reporting on the various *Anaplasma* spp. was limited to just a few organisms (cattle, sheep and goats, ticks and human beings) that were collectively conducted in a small portion of the country. This excludes a whole lot of domestic animals like horses, cats, dogs, donkeys, camels as well as wild animals such as red foxes, wild boars, deer, elk, bison, giraffes, pronghorn antelopes, and non-ruminant wildlife species like rodents, coyotes, fishers, and mountain lions; all of which are susceptible to different strains of *Anaplasma* spp. [256]. Future studies on anaplasmosis in Iran, should therefore widen their scope to include more domestic and wild animals so as to establish the host range, while ensuring that the studies cover the entire county effectively.

### Prevalence of ehrlichiosis in Iran

In the current review, a total of 11 studies reported on *Ehrlichia* spp. among dogs, cattle and ticks. The studies were only reported in 12 (38.7%) of the 31 provinces in Iran; most of them located to the northern part of the country. Given the limited number of studies and the low spatial coverage, a large portion of the country remains under researched or not researched at all. Collectively, three *Ehrlichia* species were reported from the 11 studies reviewed. The most dominant species was *E*. *canis* that was reported among dogs and ticks in 9 of the 11 studies. The pooled prevalence of *E*. *canis* among ticks (1.8–43.8%) was higher than that reported among dogs (0.8–16.6%).

*Ehrlichia canis* initially described in dogs in 1963 [260], is the primary etiologic agent of canine monocytic ehrlichiosis; a serious and sometimes fatal, globally distributed disease of dogs. The ever increasing importance of dogs as pets following continued population increase makes parasitic diseases such as *E*. *canis* a major health concern [261]. *E*. *canis* has been detected and reported in dogs from many parts of the world [262, 263].

In the current review, the highest *E*. *canis* prevalence reported among dogs was 16.6%. This was relatively lower compared to the 28% overall prevalence reported among dogs in Punjab province, Pakistan [264], 21% reported in India [265], 27% in west Indies, and 34% in Costa Rica [266]. However, the rates reported in Iran were higher than the 4.9% *E*. *canis* prevalence reported among dogs in Turkey [267]. Variation observed in the prevalence of *E*. *canis* among the various studies discussed here could be due factors such as the population density, distribution of tick vectors, the sampling methodology and the characteristics of the targeted dog population [268]. Besides ticks and dogs, studies have documented evidence of *E*. *canis* in house cats and stray cats [269]. Therefore future studies on *E*. *canis* in Iran should not only broaden their geographical range to cover the whole country but also include both house and stray cats and many other animals that may harbour these pathogens in their investigations.

In the current study, *E*. *chaffeensis* was also detected among hard ticks in Mazandaran province with a prevalence of 5.1% being reported, while an 18% *E*. *ewingii* prevalence was detected in dog’s blood films obtained randomly from different veterinary hospitals in Tehran, Iran. While studies on *E*. *chaffeensis* and *E*. *ewingii* were only conducted among ticks and dogs, respectively, in Iran, researchers in other parts of the world suggest that *E*. *chaffeensis* and *E*. *ewingii* are well known causes of human ehrlichiosis [270]. In addition, a number of domestic and wild animals are also important reservoirs of *E*. *chaffeensis* having been reported in different parts of the world. For instance, the white-tailed deer has been singled out as a major reservoir of *E*. *chaffeensis* in the United States, though the pathogen has also been detected across the globe in other deer species, such as the spotted deer in Japan and Korea and the marsh deer in Brazil, as well as in many other wild and domesticated animals [271]. Likewise, genetic materials of *E*. *chaffeensis* have been detected in coyotes and wild lemurs by PCR, while antibodies of *E*. *chaffeensis* have been reported in opossums, raccoons, rabbits and foxes in the USA [272].

From the foregoing, it is clear that studies on ehrlichiosis in Iran are still very limited and hence the need for more research focusing on a wide range of organisms and covering the whole country.

### Spotted fever group rickettsiae

In this review, only one study reported on SFG rickettsiae in a joint study conducted in four countries among them Iran [161]. The SFG rickettsiae prevalence of 45.0% by ELISA and 27.5% by IFA, reported in this review is an indication of the possibility of the presence of spotted fever group rickettsioses in Iran. The exact region or province within Iran where the study was conducted is however not given in the study. Studies conducted in southern Croatia [273], and Hungary [274] reported presence of antibodies of spotted fever group rickettsiae in domestic animals. *Rickettsia rickettsii* is regarded as the most serious species of the SFG rickettsiosis particularly in the South Atlantic and South Central census regions of the United States, where it occurs predominantly, with the *D*. *variabilis* ticks being the primary vectors in these regions [275].

This review demonstrated a complete lack of studies on SFG rickettsioses in Iran and as a result information about SFG rickettsioses in the country remains very sparse. However, absence of studies or information on SFG rickettsioses in Iran does not imply absence of the pathogen in the population and, therefore, unless studies on SFG rickettsioses are conducted among a wide range of organisms across the country, it would be difficult to establish the host range, zoonotic potential and actual prevalence of SFG rickettsioses in Iran.

### Conclusion and recommendations

This is the first review encompassing tick-borne zoonoses in the Orders Rickettsiales and Legionellales in Iran. Most studies reported on coxiellosis as opposed to anaplasmosis, ehrlichiosis and SFG rickettsioses in Iran. A large number of studies on coxiellosis relied on serological techniques (ELISA test) for antibody detection as opposed to more accurate molecular techniques. Collectively, only a limited number of organisms (cattle, sheep, goats, dogs, ticks, milk, eggs and humans) were studied, thus excluding a wide range of potential organisms particularly the wild animals that have been reported elsewhere as being susceptible to disease pathogens or harboring the various pathogens. Besides, the geographic coverage of most of these studies was very limited with most studies concentrated to the northern and southern parts of Iran. This makes it difficult to generalize the findings to the entire country.

Nevertheless, the existence of *C*. *burnetii*, *Ehrlichia* spp., *Anaplasma* spp. and *Rickettia rickettsi* in a number of organisms in 22 out of the 31 provinces in Iran as reported by various studies implies that the diseases caused by these pathogens could be highly prevalent in the country. Given that most tick-borne zoonoses are asymptomatic, there is a likelihood of silent transmission among humans in parts of the country, and thus should be considered a public health concern.

Therefore, there is need for more studies involving a wider array of organisms throughout Iran so as to establish the host-range of these tickborne zoonoses, their zoonotic potential and their actual prevalence in Iran. In addition, molecular techniques should be utilized more in the detection of pathogens and identification of the local strains that are in circulation in Iran. Active surveillance of tickborne zoonoses is therefore highly recommended as it would enable researchers to clearly define the epidemiology and public health importance of coxiellosis, anaplasmosis, ehrlichiosis and SFG rickettsioses in Iran.

## Supporting information

S1 Flow Chart The PRISMA flow chart diagram describing the articles analysis process for inclusion in the review. (DOC)

S1 Checklist Prisma 2009 check list showing checklist items against the sections, sub-sections and pages on which they appear in the document. (DOC)

S1 Accession Number Accession numbers deposited in the gene bank by various authors. (DOCX)
