# Supplementary material for: Tick-borne zoonoses in the Order Rickettsiales and Legionellales in Iran: A systematic review
Source: PLoS Negl Trop Dis. 2018 Sep 11;12(9):e0006722. doi: 10.1371/journal.pntd.0006722 (PMC6181433; doi:10.1371/journal.pntd.0006722)
Supplement: S1 Flow Chart — (DOC) [file pntd.0006722.s001.doc]

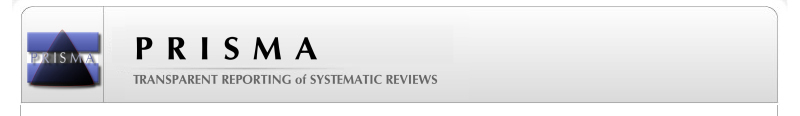
**PRISMA 2009 Flow Diagram**

**Screening**

**Included**

**Eligibility**

**Identification**

Records identified through database searching

(n = 482)

Additional records identified through other sources

(n = 723)

Total records

(n = 1205)

Records screened after removal of duplicates

(n = 952)

Records excluded

(n = 497)

Full-text articles assessed for eligibility

(n = 455)

Full-text articles excluded, with reasons

(n = 392)

Studies included in qualitative synthesis

(n = 2)

Studies included in quantitative synthesis (meta-analysis)

(n = 61)
