# Supplementary material for: Tick-borne zoonoses in the Order Rickettsiales and Legionellales in Iran: A systematic review
Source: PLoS Negl Trop Dis. 2018 Sep 11;12(9):e0006722. doi: 10.1371/journal.pntd.0006722 (PMC6181433; doi:10.1371/journal.pntd.0006722)
Supplement: S1 Accession Number — (DOCX) [file pntd.0006722.s003.docx]

**Accession Numbers**

AB196475

AF283007

EU925811

JF495135

JF495135

JF514503

JF514503

JF514504

JF514504

JF514505

JF514505

JF514506

JF514506

JF514507

JF514507

JF514508

JF514509

JF514510

JF514510

JF514511

JF514511

JF514512

JF514512

JF514513

JQ621902

JQ621902

JQ621903

JQ621903

KC685627

KC685628

KC685629

KM056396

KM056396

KM056397

KM056398

KM056399

KM056400

KM056401

KM056402

KP017262

KU242422

M60313

M73224
